# A Finite Element Stress Analysis of a Concical Triangular Connection in Implants: A New Proposal

**DOI:** 10.3390/ma15103680

**Published:** 2022-05-20

**Authors:** Romy Angeles Maslucan, John Alexis Dominguez

**Affiliations:** 1 School of Dentistry, Universidad Peruana Cayetano Heredia, Lima 15037, Peru; 2 Department of Social Dentistry, School of Dentistry, Universidad Peruana Cayetano Heredia, Lima 15037, Peru; john.alexis.dominguez@upch.pe

**Keywords:** finite element method, implant, new connection

## Abstract

Conical implant–abutment connections are popular for their stability; however, in other conditions, such as excessive force, implants and abutments can absorb all the stress. Some connections with three points of support can resist more than conical connections. In recent years, different studies has shown that the design of a connection affects its stability. The aim of this study was to analyze and compare the stresses in finite elements (FEs) in a newly proposed conical triangular connection in implants with hexagonal and conical connections. A nonlinear 3D FE parametric model was developed using SOLIDWORKS 2017^®^. All the connections, i.e., external and internal hexagons, morse taper, conical connection, and the new conical triangular proposal were compared when axial forces of 150, 250, and 350 N were applied to the occlusal. The maximum stress was found in the external hexagon. The maximum stress was concentrated at the level of the neck of the abutment, implant, and bone, except for the morse taper; at the level of the crown and abutment, the lowest stress occurred in the new proposal. Conclusions: The new conical triangular (CT) connection and the conical connection (CC) generate similar stress in the implant, abutment, and crown. However, the CT connection improves the CC by reducing stress at the bone level, adding an advantage to having three retention points.

## 1. Introduction

One of the most popular treatments for edentulous patients is dental implants. The success of supported implant prostheses is based on the integration of implants with new bone (osseointegration) [1,2]. Studies have been carried out on the influence of masticatory forces in osseointegrated implants since the 1970s, by researchers who have evaluated the functional masticatory capacity in 165 patients with implants, finding values much higher than when they used a removable prosthesis [3,4,5,6].

The biomechanics of implant rehabilitations have been well studied through experimental and FEM (finite element method) studies. These connections (between the implant and the abutment) are affected by occlusal forces; considering that, when placing implants, due to the lack of periodontal ligament, the forces are transmitted through the implant and its components as stress that will finally cause a bone resorption [7,8,9,10,11,12].

There are several types of connections (triangular, hexagons, octagons, etc.), within which the internal connections have less stress than the external connections [13,14,15,16,17,18,19,20]. Within the internal connections, the “conical” type presents better results because it generates less stress as compared with other connections, which is associated with a lower bone resorption at the implant level, as indicated by studies conducted by Coelho et al. [21], which evaluated internal and conical hexagon connections by applying 200 N and found less stress in the conical connection as compared with the hexagonal connection when subjected to vertical forces [19,20,21,22].

Connections with three points of support such as the trichannel generate better stress distribution; however, not many studies have been found in reference to these [9,10,11,12,13,14,15,16,17,18,19,20,21,22]. Studies carried out by Coppedê et al. [7], Raoofi et al. [1], and Carvalho et al. [10] among others have suggested that conical connections with three points of support such as the trichannel better distributed the stress in the implant, therefore, in this study, we evaluated a new internal connection with a triangular conical design, which could generate a reduction in stress around the bone tissue surrounding the neck of the implant [8,9,10,18,21,23,24,25,26,27,28,29,30,31,32,33,34,35,36,37,38,39,40,41,42,43].

Sakka et al. [2] conducted a literature review on the factors associated with implant failures, among which the progressive loss of marginal bone and occlusal overforce could generate alterations at the level of the abutment implant connection. Likewise, Gehrke et al. [9] and Formiga et al. [7] studied types of MEF connections: conical, internal, and external hexagon, where the implants were subjected to axial and oblique forces of 100 N, and found that the overforces occurred between the connection of the implant with the abutment, which suggested that the design of the connection could be associated with bone resorption [10,11,12].

Currently, no scientific studies that refer toa conical triangular connection; however, Raoofi et al. [1] conducted MEF studies in different types of connections (hexagon, trichannel, and conical), and found that the internal trichannel type of connection generated less stress when applying axial forces of 300 N [22].

This research proposes a conical triangular connection, which is a new proposal for implant connection to reduce the stresses transmitted to the implant. Since we know that the type of implant connection could influence the transmission of forces to the bone surrounding the implant, the conical triangular connection could achieve a decrease in saucerization (marginal bone loss of 1.5 mm during the first year and 0.2 mm annually after the insertion of the prosthesis) [8,9,10,12].

**Figure 1:**
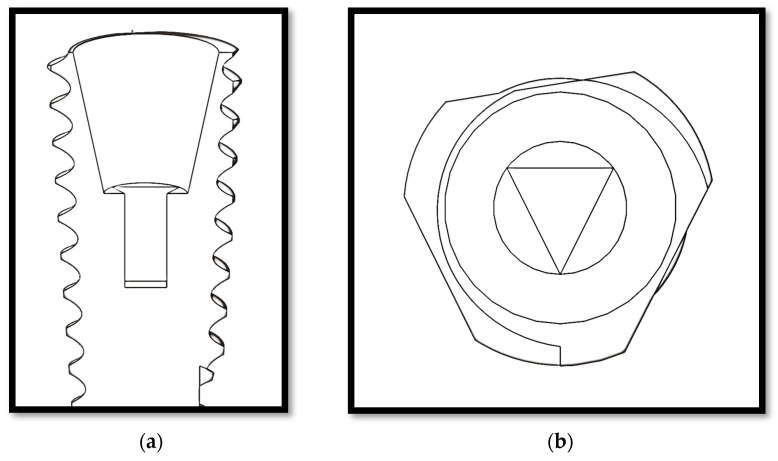
(**a**) Sagittal view of the CT connection; (**b**) occlusal view of the CT connection.

The aim of this study was to analyze and compare the effects of occlusal load direction on the stress of implant-supported partial dentures manufactured within a new connection proposal, i.e., a conical triangular (CT) (Figure 1) connection versus hexagonal, morse taper, and conical connections, with different axial forces, using a three-dimensional finite element analysis (FEA).

## 2. Materials and Methods

### 2.1. Finite Elements Model Studies

FEM studies determine through simulations the response of a body to limit situations (muscle, joint, and occlusal load) and its application in the dental field began more than a decade ago; FEM studies are currently being applied in implantology to contribute databases by analyzing the stresses that implants support during masticatory forces. In 1976, Weinstein et al. [6] were the first to use FEM in implantology. Since then, several studies have used this method to evaluate components, connections, shapes, etc. [7,8,9,10,24,41,42,43,44,45,46,47,48,49,50,51,52,53,54,55,56,57,58,59,60]. These simulations resemble reality, in such a way that their results show a high level of reliability [6,7,8,9,10].

**Figure A1:**
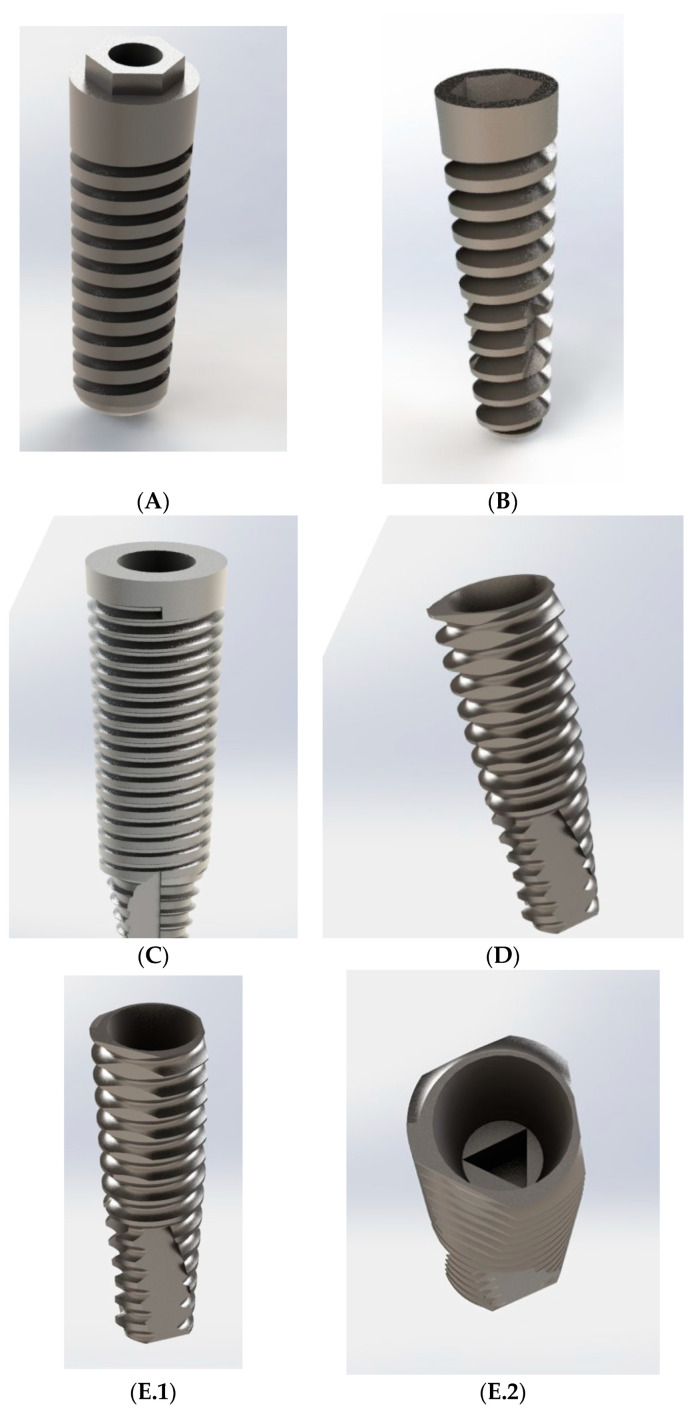
Five models of implant connections: (**A**) External hexagon; (**B**) internal hexagon; (**C**) morse taper; (**D**) conical; (**E.1**) conical triangular lateral view; (**E.2**) conical triangular occlusal view.

Three-dimensional finite elements based on solid element models were constructed that reproduced the clinical situation of an implant-supported upper premolar (SOLIDWORKS 3D CAD Design Software, Dassault Systèmes, SOLIDWORKS Corporation, Concord, MA, USA). The study group consisted of 15 models of grade 5 titanium implants with five different connection designs: external hexagon (EH), internal hexagon (IH), morse taper (MT), conical (CC), and conical triangular (CT) (Appendix A, Figure A1)). For each of the 5 implant connections, a 2 mm thick cortical bone block was modeled, and a fixed prosthesis supported by implants was designed to restore a porcelain upper premolar.

All implants were considered to be osseointegrated (bonding between the implant and surrounding bone was assumed [13]); they were designed with a diameter of 3.8 × 12 mm and placed at bone level. The implant–abutment connection systems varied for each model: EH, IH, MT, CC, and the new CT connection. Modeled prosthetic components were also integrated into the implant system. The bone–implant interface was assumed to be perfect, simulating complete osseointegration. Movement was restricted on the sides and on the lower areas, mimicking the real scenario of a dental implant placement into bone. Therefore, the connections between implant-cortical and implant-cancellous bones were designed to be bonded as well as the interface between cancellous and cortical bones. Within the implant system, FEM modeling was performed by implementing bonded conditions on the abutment–implant interfaces. The entire structure was held by setting all 6 degrees of freedom of mesiodistal surfaces of cancellous and cortical bones to zero. All the models were constructed using three-dimensional 4-node tetrahedral elements.

For all the models, the mesh type was solid standard mesh with 4 Jacobian points and quadratic elements of high order (i.e., fine mesh). The contact was global between components, and we considering that the implant was osseointegrated, the abutment fixed to the implant, and the crown was fixed to the abutment.

**Figure 2:**
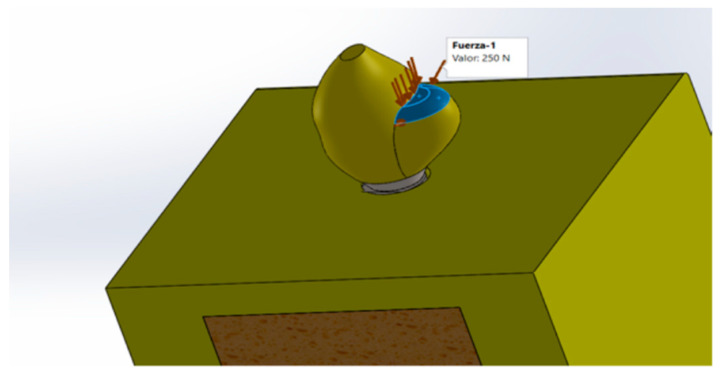
Application of forces at the occlusal side (palatal cuspid).

The boundary conditions for all models were established on both sides of the bone section. To analyze the maximum stress, axial axis of the implant forces was applied at the level of the occlusal surface of the palatine cusp (work cusp) of the upper premolar crown at forces of 150, 250, and 350 N (Figure 2).

All materials were considered to be isotropic, homogeneous, and nonlinear. Considering the physical properties of each material (Table 1), the maximum and minimum von Mises stress values were determined for each model. The von Mises analysis showed a color scale, which allowed us to visualize, in red, the areas of greatest stress. The models were labeled according to the implant–abutment connection system (i.e., EH, IH, MT, CC, and CT).

### 2.2. Data Analysis

The von Mises stresses were used to present the stress values. A stress map was generated showing the stress distribution in the implants, abutments, crowns, and bone. Because this study was a simulation using FEA, no statistical analysis was necessary [60].

## 3. Results

The highest stress values were concentrated in the neck of the implant and cortical bone, except for the MT, where the maximum stress was concentrated at the middle of the implant and the bone (Appendix B).

When analyzing the maximum stress of the crown (43.5 and 10.1 MPa, respectively, with 350 N) and the abutment, the lowest values were obtained by the CC and the new CT proposal, and the highest value was the EH, followed by the IH and MT (Table 2).

**Figure A2:**
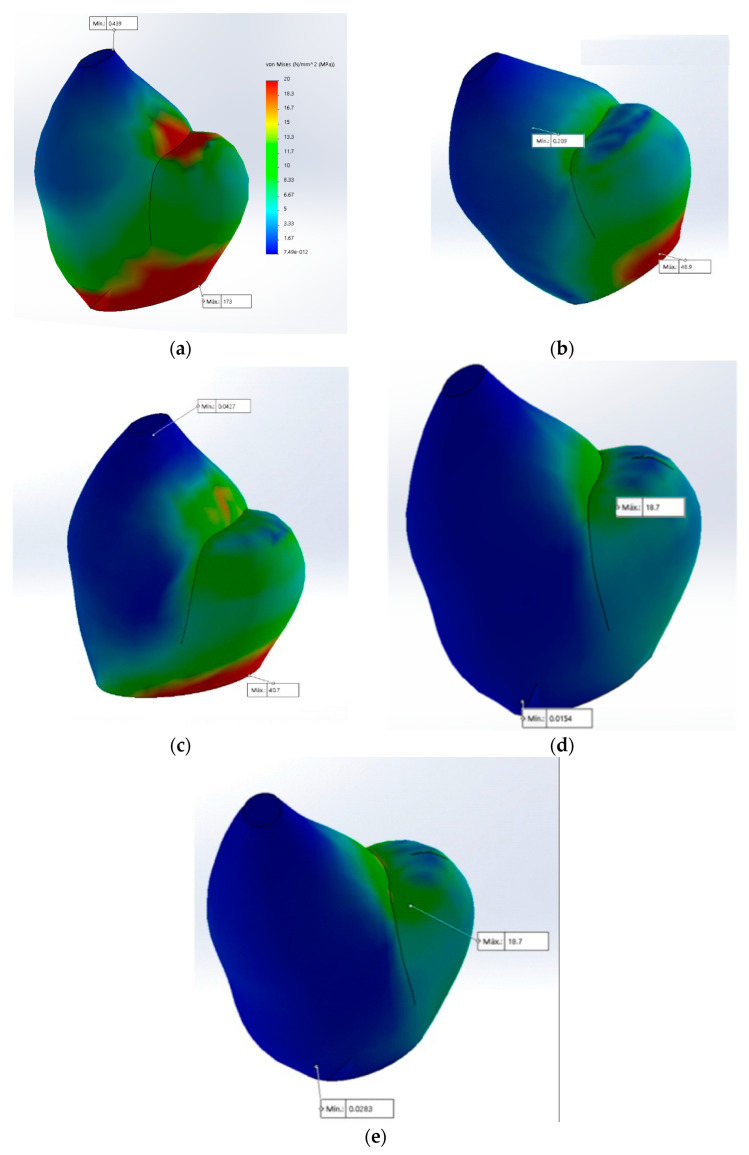
Maximun Stress in the crown with 150 N in connections: (**a**) External hexagon; (**b**) internal hexagon; (**c**) morse taper; (**d**) conical connection; (**e**) conical triangular connection.

**Figure A3:**
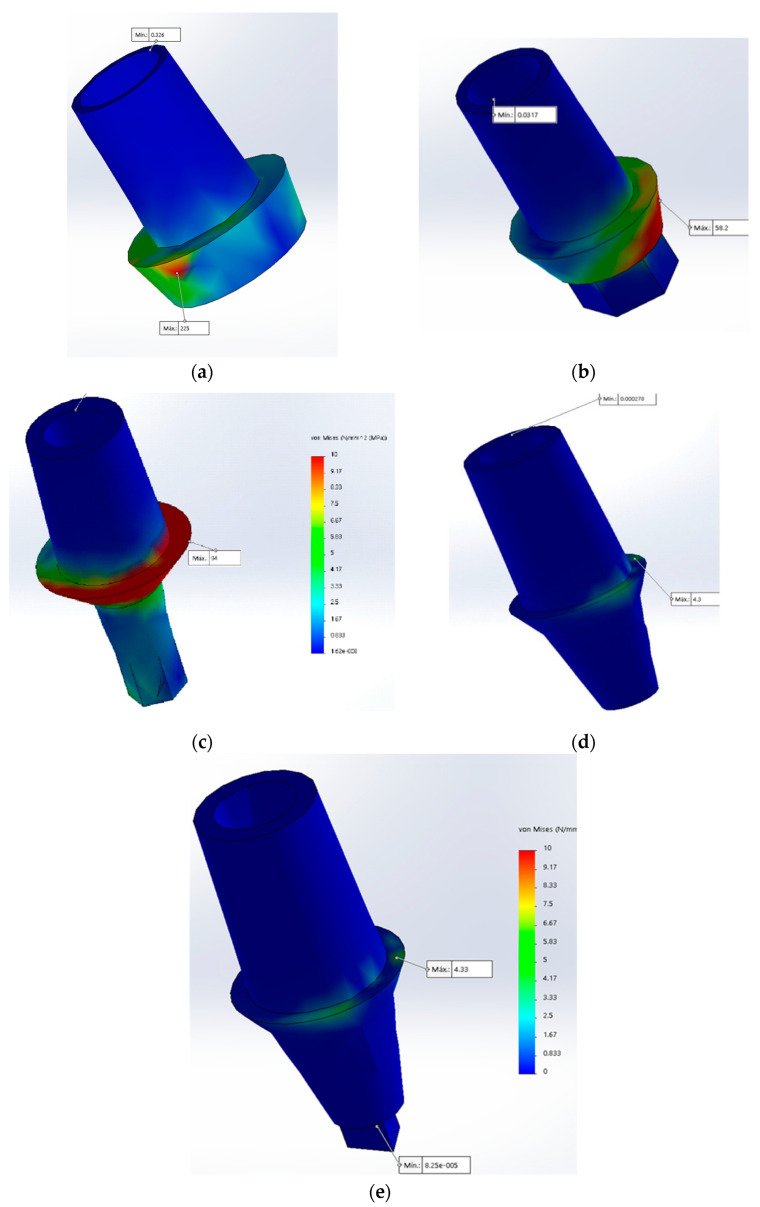
Maximun stress in the abutment with 150 N: (**a**) External hexagon; (**b**) internal hexagon; (**c**) morse taper; (**d**) conical connection; (**e**) conical triangular connection.

**Figure A5:**
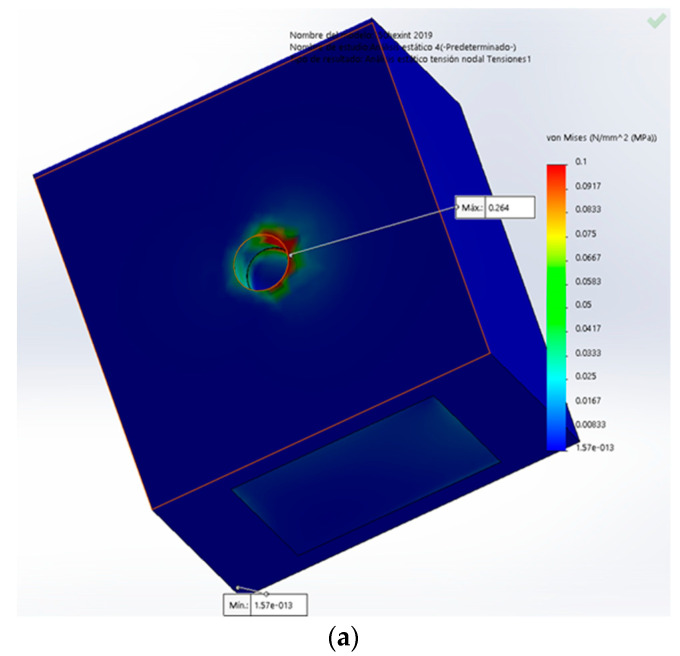

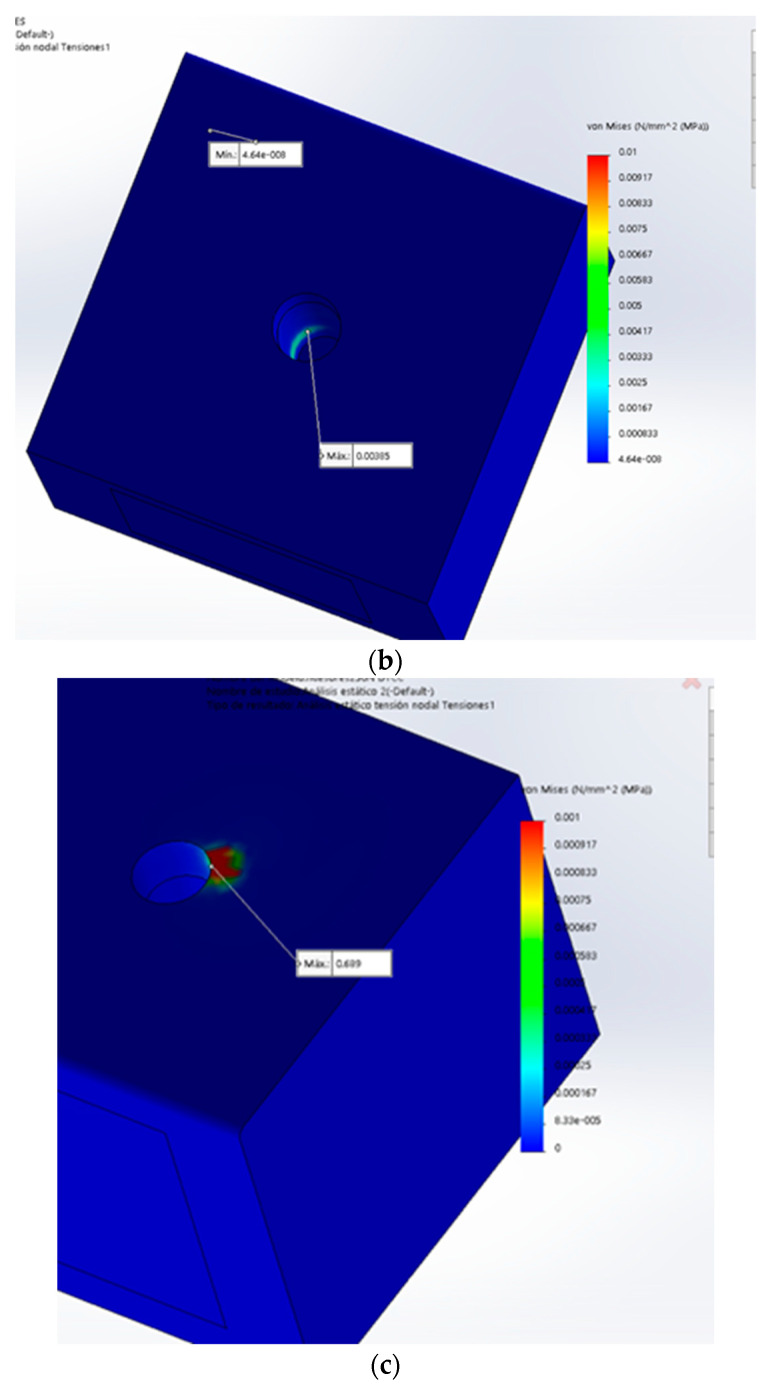

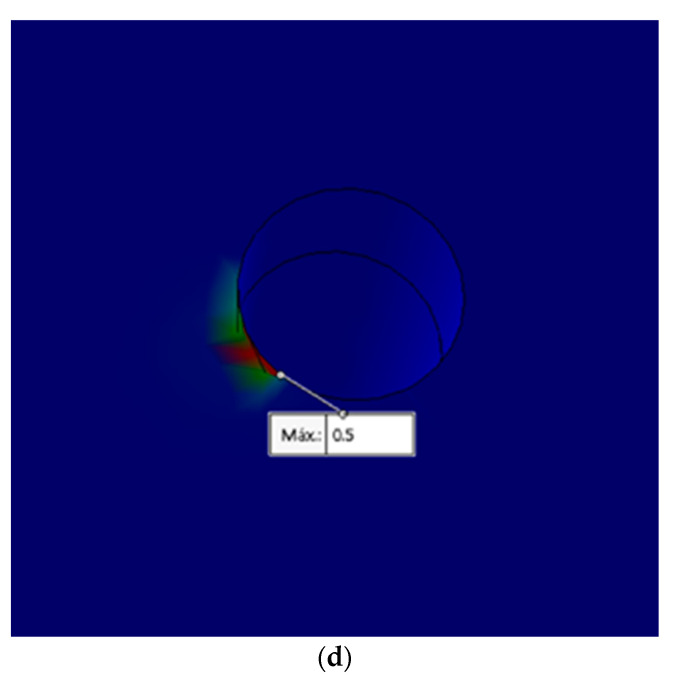
Maximun stress in the bone with 150 N: (**a**) Internal hexagon; (**b**) morse taper; (**c**) conical connection; (**d**) conical triangular connection.

At the implant level, the CC and the newly proposed CT connection generated less stress (1.49 MPa with 350 N). When analyzing and comparing the maximum stress in the MT connection applying vertical forces of 150, 250, and 350 N, the highest stresses were obtained at both the level of the crown and the abutment; however, the MT connection had the lowest stress at the bone level (Appendix B, Figure A2, Figure A3 and Figure A5).

The number of elements ranged from 42,373 to 87,618 (Table 3) (CosmosWorks, Dassault Systèmes).

### 3.1. Stress at the Crown

**Figure 3:**
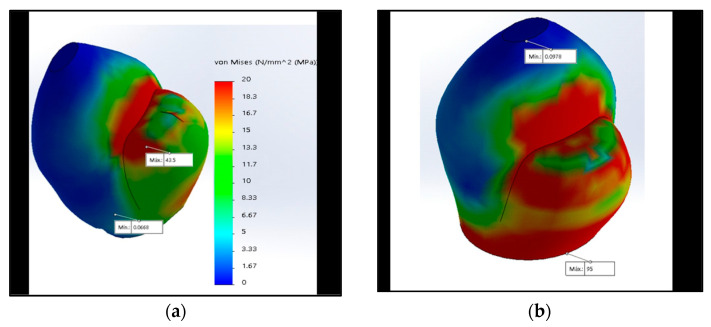
The maximum stress with 350 N at the crown is represented by red using the von Mises scale: (**a**) Maximum stress in the occlusal face in the CT connection; (**b**) maximum stress in the neck in the MT connection.

In the CC and the new CT connection, the maximum stress was located on the occlusal face (Figure 3a), unlike the IH, EH, and MT (Figure 3b), where the stress was higher and concentrated at the level of the neck’s crown.

### 3.2. Abutment

**Figure 4:**
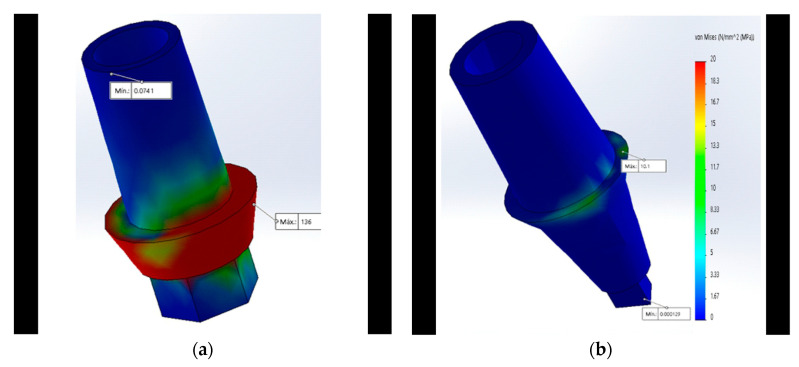
Maximum stress with 350 N at the abutment is represented by the red color using the von Mises scale: (**a**) Maximum stress at the neck in the IH connection; (**b**) maximum stress at the neck in the CT was lower (represented by the green color).

The von Mises analysis of the abutment showed that the maximum stress in the MT and the newly proposed CT was concentrated at the level of the abutment’s neck which supports the base of the crown, unlike the IH where the maximum stress was focused on the neck (Figure 4a).

### 3.3. Implant

**Figure 5:**
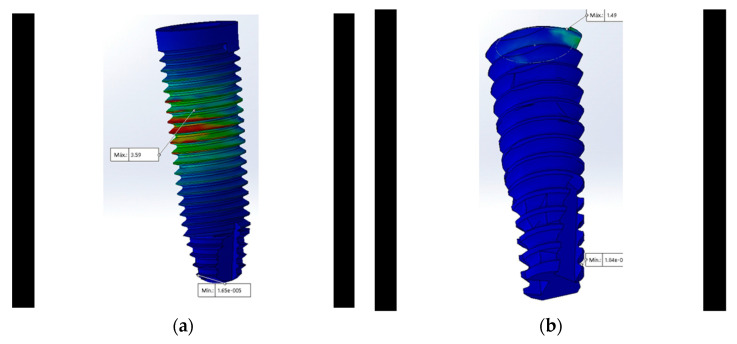
Maximum stress with 350 N in the implant is represented by the color red using the von Mises scale: (**a**) Maximum stress at the center in the MT connection; (**b**) maximum stress at the neck of the CT was lower (represented by the green color).

The von Mises analysis of the implant showed that the maximum stress in all designs except the MT (Figure 5a) was concentrated at the cervical level of the implant (Figure 5b).

### 3.4. Bone

**Figure 6:**
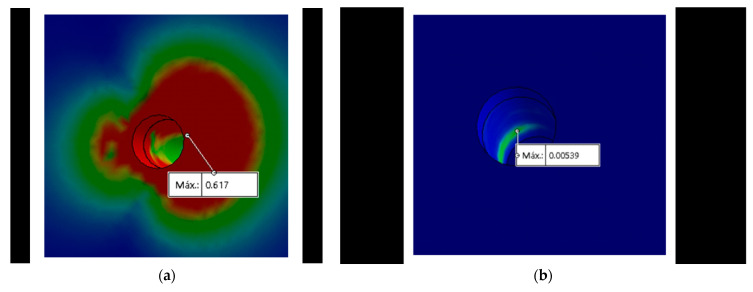

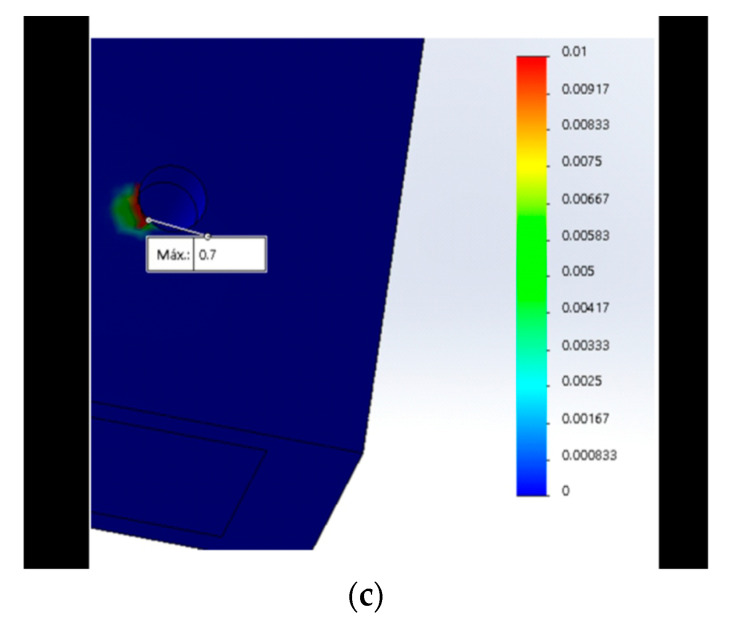
Maximum stress with 350 N in the bone is represented by the red color using the von Mises scale: (**a**) Maximum stress at the neck in the IH connection; (**b**) maximum stress at the middle in the MT connection; (**c**) maximum stress at the neck in the TC connection.

When performing the von Mises analysis in the bone, it was observed that the maximum stress in all the designs, except for the MT, was concentrated at the neck level of the bone (Figure 6a–c). In the MT connection, the stress distribution was concentrated at the middle of the bone (Figure 6b), this being the same area of the maximum concentration of stress coinciding with the MT implant (Figure 5a).

## 4. Discussion

Studies in FEM are simulations that help assess the effect of axial forces on dental implants and adjacent structures, as suggested by studies conducted by Raoofi et al. [1], Tabata et al. [10], Cho et al. [11], Borie et al. [12], Chun et al. [20], Baggi et al. [24], Quaresma et al. [25], Pessoa et al. [35], Eskitascioglu et al. [38], Siadat et al. [47], and Devaraju et al. [51], who performed analyses on FEs of implants to observe the biomechanical reactions and that of their adjacent structures. The characteristics of the number of elements (Table 3) are similar to the studies carried out by Siadat et al. [47], Raoofi et al. [1], and Cho et al. [11].

**Table 3:** Mesh characteristics.

Material	EH	IH	MT	CC	CT
Total number of nodes	61,529	102,590	120,983	120,174	127,650
Total number of items	42,373	70,200	78,599	82,528	87,618
% of distorted elements (Jacobian)	0	0	0	0	0
Item size	1.4 mm	1.16 mm	1.04 mm	1.03 mm	1.03 mm
Tolerance	0.07 mm	0.05 mm	0.05 mm	0.05 mm	0.05 mm

C. McNeill reported that, at the level of the anterior teeth, the forces received on the occlusal surface are between 100 and 150 N and, at the level of the first molar, the forces are between 300 N and 400 N on the crown [37]. Based on this, the forces of 150, 250, and 350 N were applied on the supported implant crowns.

In all designs except the MT, it was found that maximum stress was concentrated at the level of the neck of the implant and bone, which was also reflected in the implant abutments; this was comparable to studies carried out by Sutpideler et al. [57], Kitamura et al. [58], and Borie et al. [12], where they found that the highest concentration of stress was at the cervical bone and implant level.

The external hexagon:The connection that generated the greatest stress in the bone, implant, and its components was the EH. This was corroborated both by this study and studies carried out by Gehrke et al. [9] and Siadat et al. [47]. This was comparable to studies by Esposito, Tsouknidas, and Gehrke, who evaluated compressive forces and torques between external and internal hexagonal connections, and found that the EH generated greater torque and stress [9,46,47,48].

In the present study, the IH improved the external hexagon; thus, our results were similar to the studies carried out by Siadat et al. [47] and Lemus Cruz et al. [21], who evaluated microfiltration and torque and found better torque resistance to the internal hexagon connection. Although this design improved on the external hexagon, the IH generated greater stress than the newly proposed CT connection at the implant and component level.

Morse Taper: The maximum von Mises stress found in the bone was less than 0.01 MPa. This was comparable to studies conducted by Devaraju et al. and Fiorillo et al. [51], who analyzed the distribution of stress with 800 N. The results obtained with the MT connection, similar to this study, generated less stress; probably because of its morphology and conical design [7,16,21,31,32,34,47,54,59,60].

The conical Connection: The lowest stress at the level of the implants, abutment, and crown occurred in the conical connection design and in the new conical triangular (CT) connection. As shown in Table 2, this was consistent with studies conducted by Coppedê et al. [5] and [21,22], where conical connection dental implants had lower stress than the internal hexagon [25].

The Conical Triangular connection: The studies carried out by Coppedê et al., Raoofi et al. and Formiga et al., among others, suggested that conical connections in addition to three points of support, such as the proposed CT, distributed stress better in implants.

In spite of that, the CT produced similar stress to that of the CC in all components; at the bone level, even with a high value of 350 N, the stress value was 0.7 MPa (Table 2). The new CT connection generated lower stress in the bone than the CC.

The results obtained in this study provide information that helps in making decisions when evaluating the type of implant connection for patients [49], and there is less potential for fractures in the components of the CT because of its geometry (an equilateral triangle on the base of conical connection) [1].

The morse taper continues to show less stress in the bone, however, this type of connection generated more stress in both: the abutment and the crown as compared with the CC and CT connections.

Although, clinically, the internal hexagon has more positions for the abutment as compared with the new CT proposal, when we analyzed the results at the pillar level, the IH obtained more than 200% (150 N: IH, 94 MPa and CT, 4.3 MPa) and, in the crown, the maximum stress in the IH was double as compared with the CT proposal (Table 2). This justifies, from an engineering point of view, the possibility of low stress and deformation in the three-channel design due to the triangular distribution of the load over other connection designs [7,8,9], within the limitations of this study and considering the assumptions made for these FE models. It should be noted that this study was static and only analyzed the forces in work cuspid; therefore, it is recommended that future similar studies apply a study in dynamics to evaluate fatigue and, thus, observe how implant designs behave under cyclic loads.

## 5. Conclusions

The stress in the new conical triangular connection is similar tothe CC, both were lower at the level of the crown, abutment, and implant as compared with the EH, IH, and MT connections. Likewise, although the lowest stress at the bone level was in the MT connection, the maximum stress in the new CT connection was less than 1 MPa.

The CT connection as an alternative could be the first Peruvian implant connection that could compete with similar foreign implants, offering a more accessible treatment as an innovative treatment. These three support points have less potential for component fractures because of their geometry.

The results of this study present theoretical and social importance, because we evaluated the influence of design in the implant connection and its stresses distribution in the implant and the surrounding bone; and we determinated that CT and CC are the most conservative and longevity treatment option for our patients.patients.

Although the hexagon has more possibilities of positions for the abutment, at the level of the implant, the hexagon generates more stress than the conical connection and the conical triangular connection. Taking into consideration the finding by Borie et al. [14] who showed that when a load was applied to an implant it was partially transferred to the bone, CT could be an option for patients with parafunction such as bruxism.

The present study shows that the new CT connection is viable, since it presents lower stress on the implant with similar values to that of CC; both connections obtain less stress in the abutment and crown than conventional connections. Furthermore, the CT conneciton improves the CC at bone level. For this reason, it is an alternative for both specialists in implantology and dental prosthetists.

## 6. Patents

This new conical triangular connection has a patent in Peru and is also published in Google patents. https://patents.google.com/patent/PE20210771Z/en?q=romy+angeles&oq=romy+angeles (accessed on 24 March 2022).

## Figures and Tables

**Table 1:** Mechanical proprieties used in finite element analyses.

Material	Young’s Modul (MPa)	Poisson’s Ratio (v)	Reference(s)
Porcelain	68,900	0.28	De Carvalho Formiga et al. [9]
Titanium Alloy (Ti–6Al–4V)	110,000	0.35	De Carvalho Formiga et al. [9]; Yao, K.-T. et al. [61]
Cancellous Bone	1370	0.30	De Carvalho Formiga et al. [9]; Yao, K.-T. et al.
Cortical Bone	13,700	0.30	Yao K-T et al. [61]

**Table 2:** The von Misses stress in the connections.

Component	Crown			Abutment		
Forces	150 N	250 N	350 N	150 N	250 N	350 N
EH	173	288	404	255	375	525
IH	48.9	81.5	114	58.2	97	136
MT	40.7	67.8	95	94	157	219
CC	18.7	31.1	43.5	4.3	7.17	10
CT	18.7	31.1	43.5	4.3	7.21	10.1
**Component**	**Implant**			**Bone**		
**Forces**	**150 N**	**250 N**	**350 N**	**150 N**	**250 N**	**350 N**
EH	75	125	175	75.1	125	175
IH	11.9	19.8	27.8	0.26	0.44	0.617
MT	1.54	2.56	3.59	0.002	0.003	0.0053
CC	0.19	0.33	0.465	0.41	0.68	0.964
CT	0.63	1.06	1.49	0.3	0.5	0.7

EH, external hexagon; IH, internal hexagon; MT, morse taper; CC, conical connection; CT, conical triangular (new connection).

**Figure A4:**
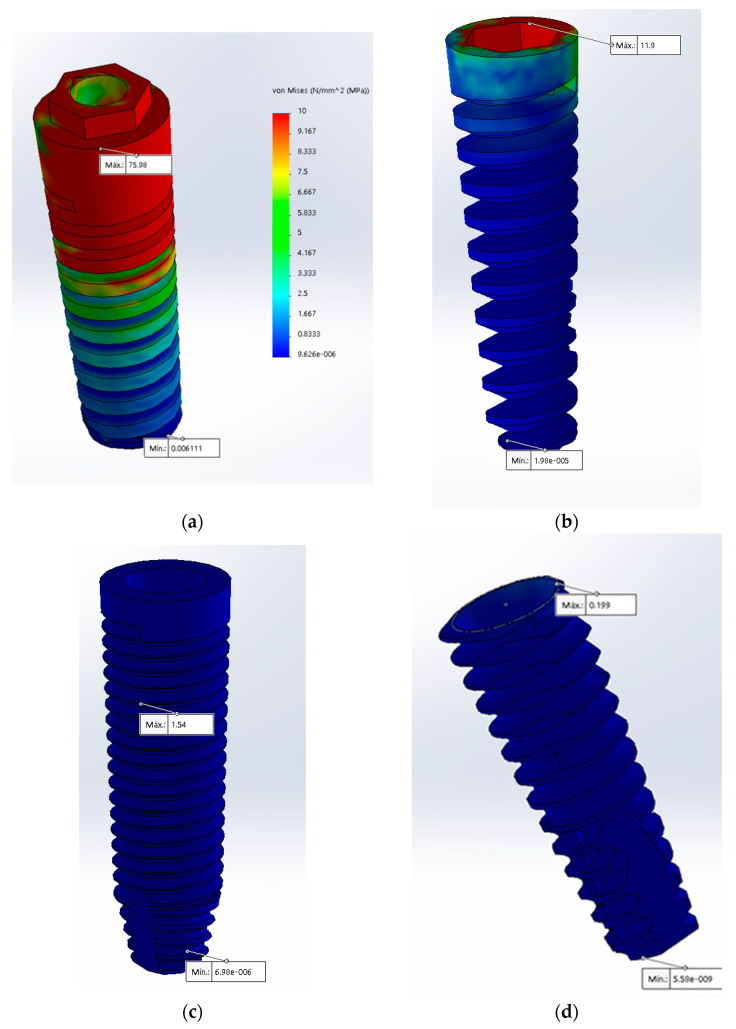

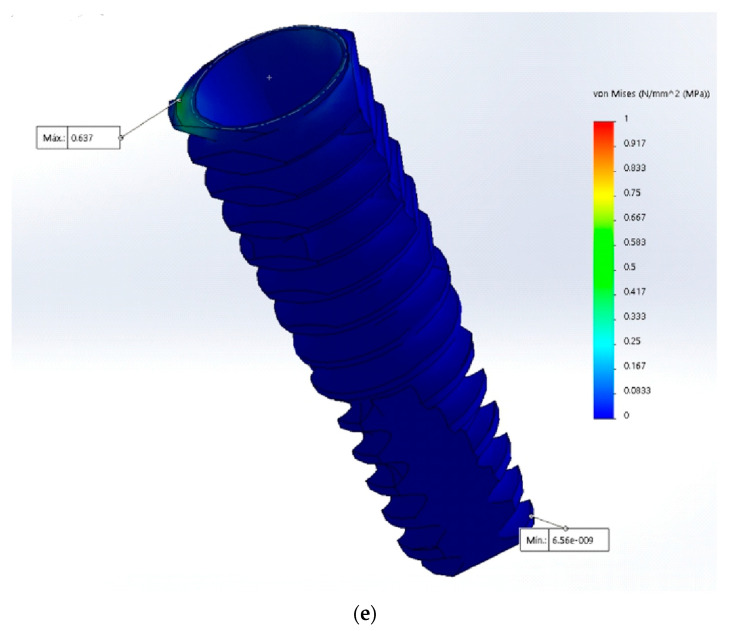
Maximun stress in the implant with 150 N: (**a**) External hexagon; (**b**) internal hexagon; (**c**) morse taper; (**d**) conical connection; (**e**) conical triangular connection.

## Data Availability

The data is available on https://repositorio.upch.edu.pe/handle/20.500.12866/10027.
